# The Clinical and Immunological Activity Depending on the Presence of Interferon γ in Primary Sjögren’s Syndrome—A Pilot Study

**DOI:** 10.3390/jcm11010003

**Published:** 2021-12-21

**Authors:** Agata Sebastian, Marta Madej, Maciej Sebastian, Anna Łuczak, Paweł Gajdanowicz, Magdalena Zemelka-Wiącek, Piotr Wiland

**Affiliations:** 1 Department of Rheumatology and Internal Medicine, Wroclaw Medical University, 50367 Wroclaw, Poland; agatasebastian@vp.pl (A.S.); woochi@wp.pl (A.Ł.); pwiland1@gmail.com (P.W.); 2 Department of General, Minimally Invasive and Endocrine Surgery, Wroclaw Medical University, 50367 Wroclaw, Poland; mseba@op.pl; 3 Department of Clinical Immunology, Wroclaw Medical University, 50367 Wroclaw, Poland; pawel.gajdanowicz@umed.wroc.pl (P.G.); magdalena.zemelka-wiacek@umw.edu.pl (M.Z.-W.)

**Keywords:** primary Sjögren syndrome, interferon γ, disease activity

## Abstract

The upregulation of IFN pathways and their stimulated genes is associated with primary Sjögren’s syndrome (pSS). The recent studies also indicate the involvement of interferon γ (IFNγ) in the pathogenesis of pSS. The study aimed to assess the clinical and immunological activity depending on the concentration of IFNγ in the peripheral blood in pSS patients. Methods: The study group consisted of patients over 18 years of age with a confirmed diagnosis of pSS. Based on the collected data, disease activity was assessed using the EULAR Sjögren’s syndrome disease activity index (ESSDAI) and the EULAR Sjögren’s syndrome patient reported index (ESSPRI). Results: Among 40 pSS patients, 33 (82%) showed increased levels of IFNγ. The group with positive IFNγ was younger (43 years) than the group with negative IFNγ (57 years) (*p* < 0.05). In the positive IFNγ group, the time to diagnosis was shorter (*p* < 0.05). There was a difference in ESSDAI among patients with and without IFNγ (*p* < 0.05). There were no differences between the groups in ESSPRI and the presence of cryoglobulins, specific anti-SSA, and anti-SSB antibodies and in C3 and C4 hypocomplementemia. RF occurred in both groups with a similar frequency (*p* = 0.6), but in patients with IFNγ presence, significantly higher RF titers were observed (34.9 vs. 10.5; *p* < 0.05). Conclusion: In the group of patients with positive IFNγ, the mean value of RF and ESSDAI was higher. This group was also younger than patients with pSS without IFNγ.

## 1. Introduction

Sjögren’s syndrome (SS) is the second most common autoimmune disorder after rheumatoid arthritis (RA) [1]. Primary SS (pSS) is considered a multifactorial disease, where a susceptible genetic background requires an environmental factor trigger, such as viral infection [2], to initiate the development of the disease. Many genes, including IRF5, STAT4, and IL12A, regulate the interferon (IFN) system [3,4]. The upregulation of IFN pathways and their stimulated genes are associated with the clinical symptoms of SS [5,6]. There are three main groups of IFN: type I, which includes various forms of IFNα and one IFNβ; type II IFN charge IFNγ; and type III IFN-λ consisting of IL-29, IL-28A, and IL-28B. The studies conducted so far among patients with pSS indicate that IFN may play a significant role in the pathogenic mechanism and development of clinical symptoms. In the process of disease initiation, IFN I is the critical factor that activates many genes located in the so-called signature for IFN [7,8]. Type I IFN belongs to the proteins involved in antiviral protection. Dendritic cells are the main source of the cytokine. Virus-infected cells can be destroyed by NK cells, which are enhanced by IFN type I. In the sera of pSS patients compared to healthy individuals, active levels of IFN type I were observed more frequently [9,10].

However, recent studies also indicate involvement in the pathogenesis of pSS IFN type II (IFN γ) and it was confirmed in both animal models and human studies [8,11,12,13,14,15]. IFNγ is mainly produced by NK cells and T lymphocytes, and to a lesser extent, by dendritic cells, macrophages, and B lymphocytes [16]. After binding to the IFNγ receptor, IFN II signature genes are induced, promoting antibacterial protection, inflammation, and tissue damage [17,18]. In a study by Nezos et al. [7], the researchers demonstrated a signature for IFN I in the sera of pSS patients and IFN type II in the histopathological material of the minor salivary glands.

Additionally, in biopsies of salivary gland tissues collected from pSS patients with lymphomas, a lower transcription of IFNα, but higher IFNγ was observed more often [7]. Considering the above, it appears that blocking IFN may be one of the therapeutic options in pSS. As is the case with systemic lupus erythematosus, in therapy, there are attempts to use sifalimumab [19], rontalizumab (antibodies that bind to IFN), and the kinoid IFNα [20]. In addition, there is a significant correlation between IFNα and BAFF (B-cell activating factor) because BAFF expression is directly induced by type I IFN, suggesting that blocking IFN type I can reduce BAFF expression and thus reduce autoreactivity of B cells and antibody production [21]. It was also shown that cells on the ocular surface in pSS patients are susceptible to the action of IFNγ. Therefore, drugs targeting this cytokine could increase the synthesis and secretion of mucin on the eye surface [22].

So far, however, we do not have many studies assessing the influence of interferon on the occurrence of specific organ changes in the course of pSS.

Our study aimed to assess the clinical and immunological activity depending on the concentration of IFNγ in the peripheral blood in pSS patients.

## 2. Materials and Methods

The study group consisted of patients over 18 years of age with a confirmed diagnosis of pSS based on the current 2016 ACR-EULAR Classification Criteria from 2016 [23], hospitalized in the Rheumatology Department between 2015 and 2020.

No participant had evidence of lymphoma, sarcoidosis or hepatitis C, another connective tissue disease diagnosis, asthma, allergy or infectious diseases (in this viral infection) in the last three months. None of the patients used nonsteroidal anti-inflammatory drugs 72 h before blood was drawn for laboratory tests. After obtaining written consent, a detailed medical history was received for all patients, a physical examination was performed, and blood was collected for laboratory determinations. Based on the collected data, disease activity was assessed using the EULAR Sjögren’s syndrome disease activity index (ESSDAI) [24] and the severity of dryness, fatigue, and pain using the EULAR Sjögren’s syndrome patient reported index (ESSPRI) [25].

### 2.1. Serum Sampling

Ten milliliters of venous blood were taken from the antecubital vein by a standard venipuncture method. One part of the serum was separated from blood by centrifugation and then stored at −80 °C until subsequent biochemical analyses of IFNγ and thawed immediately before assay. Serum cytokines concentration was evaluated using LEGENDplex (Biolegend) kits. The analysis used the BD CANTO2 flow cytometer and LEGENDplex data analysis software suite, according to the manufacturers manual. The minimum detection limit for IFNγ was 36.57 pg/mL.

Antinuclear autoantibodies (ANAs) were determined in the second part of the serum and quantified using indirect immunofluorescence technology (Hep2 cells). Titers of 1:320 or higher were considered positive. Anti-Ro/SSA and anti-La/SSB autoantibodies were detected by an enzyme-linked immunosorbent assay (ELISA) for the quantitative determination of IgG autoantibodies (EUROIMMUN). Quantitative determination of serum rheumatoid factor (RF) was performed using an immunonephelometry test. Additionally, blood morphology, C-reactive protein (CRP), erythrocyte sedimentation rate (ESR), electrolytes, cryoglobulins, C3 and C4 complement components, electrophoresis, and the concentrations of IgG and IgM were measured in all patients. Each patient was also analyzed for a urine sample for abnormalities in its composition.

The study was performed according to the principles of the Declaration of Helsinki and was approved by the local ethics committee; written informed consent was obtained from all the participants. The Medical University’s ethics committee approved the study (decision number 390/2015).

### 2.2. Statistical Analysis

The analysis was performed using Statistica 10 software package.

The Mann–Whitney U test was used to compare the distributions of quantitative variables in two independent groups. The chi-squared test was used to verify the relationships between dichotomous variables. Significance was reported at a value of *p* ≤ 0.05.

## 3. Results

The study group consisted of 40 patients diagnosed with pSS (37 women and 3 men). Most of the patients showed elevated levels of IFNγ (33 patients, 82% of patients)—the group with positive IFNγ (concentration of IFNγ > 36.57 pg/mL). In 7 patients, no increased levels of IFNγ were observed (18% of patients)—the group with negative IFNγ (concentration of IFNγ ≤ 36.57 pg/mL). The group with positive IFNγ was statistically younger (43.5 years) than the group with negative IFNγ (57 years) (*p* < 0.05). In the positive IFNγ group, the time to diagnosis was significantly shorter, on average by 12 months (*p* < 0.05). The mean follow-up time from the diagnosis of pSS to the determination of IFNγ was four and a half years (in the entire analyzed population). There was a statistically significant difference in pSS activity (assessed using the ESSDAI index) among the group of patients with and without IFNγ (*p* < 0.05). However, in any of the domains constituting the ESSDAI, no statistically significant difference was found among the studied groups of patients. In the ESSPRI questionnaire, greater severity of the reported dryness symptoms was found in the IFNγ negative group than in the positive group (6.1 vs. 4.6). A similar relationship was observed concerning the severity of fatigue (mean 6.1 vs. 4.8). However, these values were not statistically significant (*p* = 0.1 and 0.3). There were no differences in the presence of cryoglobulins, specific anti-SSA, and anti-SSB antibodies, in C3 and C4 hypocomplementemia among the groups. RF occurred in both groups with a similar frequency (*p* = 0.6), but in the group of patients with IFNγ presence, statistically, significantly higher RF titers were observed (34.9 vs. 10.5; *p* < 0.05). All marked laboratory parameters and the components of ESSDAI and ESSPRI are presented in Table 1, Table 2 and Table 3.

Pharmacological treatment included hydroxychloroquine (in 50% of patients) in a similar percentage in the group with and without IFNγ and azathioprine (*p* = 0.8). In the group of patients with IFNγ, methotrexate, cyclosporine, and mycophenolate mofetil were also used (Table 4). No relationship was observed between disease duration and IFNγ levels (*p* = 0.14), regardless of the immunosuppressive treatment used (mean IFN γ level < 5-year duration was 257 pg/mL, ≥5 years 205 pg/mL).

According to sex, males were younger than females (29 vs. 49 years), and anti-SSA and anti-SSB antibodies were found in all of them. The RF was present in most men (two of three) and had lower mean titers than women (20 vs. 36.6 IU/mL). The ESSDAI value was higher in women than men (9 vs. 7 points), as were the individual components of the ESSPRI-dryness symptoms (5.3 vs. 4.6 points), fatigue (5.2 vs. 4.6 points) and pain (4.0 vs. 3.0 points). Women were also more likely to use immunosuppressive drugs, such as azathioprine, cyclosporine, mycophenolate mofetil. The presence of IFNγ in serum was detected in all male patients, but because of the very small group size (only three men), we cannot draw firm conclusions.

## 4. Discussion

pSS is a very heterogeneous disease that may involve multiple organs. The course of the disease is different in each patient and requires an individual approach. We currently distinguish two main phenotypes of the disease-with the predominance of dryness or organ involvement symptoms.

Recently, many attempts were made to assess the pathophysiological pathways determining organ involvement in pSS, based on thorough genetic studies [26]. To this day, however, we do not know the key biomarkers of the disease, the determination of which would allow for predicting the phenotype of the disease and personalizing therapy at its early stages. In our work, we present the relationship between the serum concentration of IFNγ in patients with diagnosed pSS and the clinical course and immune picture of the disease.

In line with our original hypothesis, we confirmed that patients with higher disease activity expressed in ESSDAI have an increased concentration of IFNγ, which affirms that IFNγ is one of the factors involved in the pathogenesis of pSS. An important observation was that the group of patients with elevated IFNγ was statistically younger (on average by ten years) and required a shorter time to complete the diagnosis of pSS from the moment of the first symptoms.

Studies to date indicate that over half of all pSS patients exhibit an IFN signature, and these patients typically present higher values of disease activity (immunological and clinical) [27]. Our observation results confirmed that increased interferon levels occurred in more than ¾ of the analyzed group of patients (33 out of 40 subjects). Similar to the previous study results, we also observed a relationship between higher IFN concentrations and higher ESSDAI values. However, in our study, we did not find statistically significant differences between the individual components of ESSDAI. To finally verify the possible association of IFNγ with the effect on specific organ complications of pSS, larger groups of patients should be compared.

Similarly, Hall and Bodewes et al. [28,29,30] demonstrated that high IFN activity was associated with a more severe disease phenotype in pSS. In the cited articles, however, in contrast to us, in the group of patients with higher IFN expression in laboratory tests, higher ANA titers, more frequent presence of anti-SSA/SSB antibodies, higher IgA concentrations, and leukopenia were noted [28,29,30]. Higher levels of IFN-induced protein expression were evident in participants with greater salivary gland focus scores > 3 [28].

In our population, the group of patients with elevated serum IFNγ concentration did not differ significantly from the group with negative determination in terms of the presence of ANA, specific antibodies, hematological domain, and focus score. However, we noticed that although RF was equally common in both analyzed subgroups of patients (IFNγ positive vs. IFNγ negative), higher RF titers were found in IFNγ positive patients, which is a new observation. Indirectly, this could mean that patients with the higher serological activity of pSS do have elevated IFNγ. High-affinity RF, produced by CD5+ B cells, is produced by prolonged stimulation of the immune system. Therefore, it can exacerbate the autoimmune response. Increased titer RF appears to be important for the assessment of pSS activity, as its presence was associated with a more severe course of pSS and correlated with the production of gammaglobulins and other autoantibodies, including anti-SSA and anti-SSB [31]. It is also suspected that RF overproduction in pSS may be associated with a risk of lymphoma development [32].

Recent advances in basic research have increased our understanding of the dry eye disease caused by SS. It was reported that IFN activates various cytokines produced by immunocompetent cells, including IFN, interleukin (IL)-17, and autoreactive T and B cells in the immune pathogenesis of SS exocrine glands [33,34]. In animal models, it was shown that IFNγ is associated with more significant destruction of the tear gland tissue, which results in lower tear production [35]. In our group of pSS patients, dryness symptoms expressed by the ESSPRI were less pronounced in younger patients with elevated IFNγ levels. However, this difference was not statistically significant.

The limitation of our work is the lack of a control group and the lack of determination of the baseline IFNγ concentrations at the time of pSS diagnosis. Additionally, immunosuppressive medications were not stopped before serum measurements were made, and a potential effect on the serum IFNγ concentration cannot be ruled out. In the study group we analyzed, half of the patients used hydroxychloroquine. However, it is worth noting that there should be a trend toward lower serum IFN levels in patients receiving antimalarial drugs. This conclusion could be based on the recently published JOQUER study results in which the authors demonstrated a reduction in IFN I in patients treated with hydroxychloroquine [36]. However, in our study, we determined IFNγ (IFN type II) and not IFN type I. It may indicate the different roles of IFN types I and II in the pathogenesis of pSS. While the pathogenesis of SS is not well understood, both innate and adaptive immune responses are implicated in disease initiation and progression. In the innate response, an antiviral response is mounted through the recognition of viral nucleic acids by Toll-like receptors (TLRs). This recognition leads to the upregulation of the type 1 IFN pathway. However, the means by which immune activation is initiated and maintained remain incompletely understood. Plasmacytoid dendritic cells (pDC) are the premier IFN I producing cells. pSS pDCs produced higher levels of pro-inflammatory cytokines, including type-I IFN, upon in vitro stimulation with endosomal Toll-like receptor ligands [36,37].

On the other hand, immunofluorescent analysis in salivary sections from pSS patients showed that C-X-C motif chemokine 10 (CXCL10) and matrix metalloproteinase 9 (MMP9) were strongly co-expressed in expanded ductal cells and were associated with the presence of infiltrating immune cells around expanded ducts. In contrast, acinar cells did not express CXCL10 and MMP-9. MMP-9 inhibition could suppress the CXCL10 expression in human salivary gland ductal cells via a decrease in STAT1 phosphorylation and, therefore, IFNγ signaling [38]. Other authors showed that the absence of IL-27 signaling (an immunomodulatory cytokine mainly produced by resident myeloid cells) led to an uncontrolled Th17 expansion in the glands, which in turn fostered an exaggerated expansion and aberrant activity of salivary glands (ectopic lymphoid structures). In SS, they found that despite pSS patients expressing high local and peripheral levels of IL-27, this cytokine was unable to inhibit Th17 differentiation and conversely induced a strong IFNγ response compared to healthy donors [39,40].

## 5. Conclusions

There is a long-lasting need for non-invasive, more accurate diagnostic techniques to evaluating pSS patients. Incorporating additional diagnostics involving screening for disease-specific biomarkers in biological fluid is a promising concept that requires further investigation. IFNγ may be one such biomarker.

Previous studies have clearly shown that pSS patients display higher expression of type I and type II IFN-regulated genes in both the affected salivary tissue and peripheral blood. Although the numbers in our groups are small, it is noteworthy that the differences in RF value and ESSDAI reached statistical significance.

In the group of patients with positive IFNγ, the mean value of RF and ESSDAI was higher. This group was also statistically younger than patients with pSS without IFNγ. The results of our study also indicate that the group of patients with positive IFNγ require more intensive immunosuppressive treatment, including the use of drugs such as methotrexate, mycophenolate mofetil, and cyclosporine. However, this is only a hypothesis, as the analyzed patient groups (IFNγ positive vs. IFNγ negative) differed significantly in terms of numbers (33 vs. 7). Hydroxychloroquine and azathioprine were used in a similar percentage of patients in both analyzed populations. Although the era of biologic treatment modalities has not led to any approvals for pSS, the arrival of JAK inhibitors in RA (tofacitinib and baricitinib), with additional compounds at late stage or close to approval (upadacitinib and filgotinib), suggests that the potential of these JAK inhibitors to target IFN pathways (at least partially type I and II IFN) is of particular interest in pSS [41].

## Figures and Tables

**Table 1:** Characteristics of pSS patients with positive and negative IFNγ in peripheral blood.

Parameter	IFNγ Negative Group (*n* = 7)	IFNγ Positive Group (*n* = 33)	*p*-Value
Age of patients (years)			
- mean (SD)	57.7 (12.0)	43.5 (13.3)	0.04 (test U)
Time from pSS diagnosis (years)	5.2 (3.5)	4.27 (4.28)	0.01 (test U)
ESSDAI value			
- Mean (SD)	6.2 (2.0)	10.4 (6.2)	<0.05(test U)
C3 hypocomplementemia (*n*)	1	4	0.8 (test X^2^)
C4 hypocomplementemia (*n*)	1	5	0.9 (test X^2^)
Positive anti-SSA antibodies (*n*)	6	26	0.8 (test X^2^)
Positive anti-SSB antibodies (*n*)	4	20	0.9 (test X^2^)
Focus score ≥ 1 (*n*)	4	30	0.4 (test X^2^)
Rheumatoid factor (nv < 14 IU/mL)			
- Positive results (*n*)	3	28	0.3 (test U)
- Mean (SD)	10.5 (11.4)	34.9 (72.1)	<0.05 (test X^2^)
- median	5	9	
Positive cryoglobulins (*n*)	1	0	-
ESR mean value (nv 3–15 mm/hr) (SD)	11 (4.9)	17.8 (13.3)	0.1 (test U)
CRP mean value (nv 0–5 mg/L) (SD)	0.8 (0.5)	1.8 (2.7)	0.1 (test U)

Abbreviations: IFN—interferon γ; SD—standard deviation; ESSDAI—the EULAR Sjögren’s syndrome disease activity index, *n*—number of patients; nv—normal value; ESR—erythrocyte sedimentation rate; CRP-C-reactive protein.

**Table 2:** The point value of individual ESSDAI domains in pSS patients with positive and negative IFNγ concentration in peripheral blood.

ESSDAI Domain (Weight > 0)	IFNγ Negative Group Number of Patients	IFNγ Positive Group Number of Patients	*p*-Value
Constitutional	0	4	-
Lymphadenopathy	0	2	-
Lymphoma	0	0	
Glandular	3	6	0.2 (test X^2^)
Articular	4	22	0.8 (test X^2^)
Cutaneous	0	4	-
Pulmonary	2	6	0.6 (test X^2^)
Renal	0	1	-
Muscular	0	0	-
Peripheral nervous system	0	0	-
Central nervous system	0	0	-
Hematological	2	8	0.8 (test X^2^)
Biological	3	16	0.8 (test X^2^)

Abbreviations: IFN—interferon γ; ESSDAI—the EULAR Sjögren’s syndrome disease activity index, *n*—number of patients.

**Table 3:** The point value of individual ESSPRI domains in pSS patients with positive and negative IFNγ concentrations in peripheral blood.

ESSPRI Score (Min 0 to Max 10 Points)	IFNγ Negative Group (0–10 Points)	IFNγ Positive Group (0–10 Points)	*p*-Value
Dryness–mean (SD)	6.1 (4.1)	4.6 (2.2)	0.1
Fatigue–mean (SD)	6.1 (3.0)	4.8 (2.2)	0.3
Pain–mean (SD)	4.0 (3.5)	3.0 (2.6)	0.5

Abbreviations: IFN—interferon γ; ESSPRI—the EULAR Sjögren’s syndrome patient reported index.

**Table 4:** The treatment used in pSS patients depending on the concentration of IFNγ.

Medicament	IFNγ Negative Group Number of Patients	IFNγ Positive Group Number of Patients	*p*-Value
Hydroxychloroquine	3	17	0.06 (test X^2^)
Methotrexate	0	5	-
Azathioprine	1	4	0.08 (test X^2^)
Cyclosporine	0	2	-
Mycophenolate mofetil	0	1	-
Combination therapy	1	10	0.4 (test X^2^)
No therapy	3	4	0.1 (test X^2^)

## Data Availability

All data are archived at the Department of Rheumatology and Internal Medicine (Medical University of Wrocław, Poland) and can be provided by the authors-Agata Sebastian, Marta Madej and Piotr Wiland.
